# Advancing Multimodal Medical Image Generation: A Self‐Improving Generative Foundation Model

**DOI:** 10.1002/mco2.70490

**Published:** 2025-12-14

**Authors:** Yongjian Chen, Lui Ng

**Affiliations:** ^1^ Dermatology and Venereology Division Department of Medicine Solna Center for Molecular Medicine Karolinska Institute Stockholm Sweden; ^2^ Department of Surgery School of Clinical Medicine Li Ka Shing Faculty of Medicine The University of Hong Kong Hong Kong China

1

A recent study published in Nature Medicine introduces a unified medical image‐text generative model (MINIM) that addresses the longstanding challenge of limited high‐quality medical imaging datasets [1]. Generative artificial intelligence (AI) models like MINIM represent a promising advance that may help researchers and clinicians address data limitations and accelerate the development of AI‐driven medical tools.

In clinical and research settings, acquiring large, diverse, and well‐annotated medical imaging datasets remains a critical challenge. This scarcity is especially pronounced for rare diseases, emerging imaging technologies, and underrepresented patient populations, where data collection is limited by small sample sizes, privacy concerns, and legal restrictions on data sharing [2]. As a result, the development and generalizability of AI applications in healthcare are often constrained, limiting their potential for improving diagnosis and treatment. By synthesizing medical images across various imaging modalities and anatomical sites from textual instructions, MINIM offers a potential approach for augmenting existing datasets.

MINIM is a versatile, self‐improving generative AI model notable for its multimodal capabilities. Unlike conventional image‐text generative models, which are typically restricted to a single imaging modality [3], MINIM can generate high‐quality synthetic images across a variety of modalities. During its development, the authors trained MINIM using a diverse set of medical images, including optical coherence tomography (OCT), fundus photography, chest X‐ray, and chest computed tomography (CT), with each image paired with a corresponding textual description. This training strategy allowed the model to embed rich medical image‐text knowledge into a stable diffusion framework (Figure 1A). During inference, MINIM generates synthetic images directly from text prompts (Figure 1B). By effectively combining images with their paired textual information, the model produces outputs that resemble real clinical data, potentially mitigating data scarcity in medical AI applications.

**FIGURE 1:**
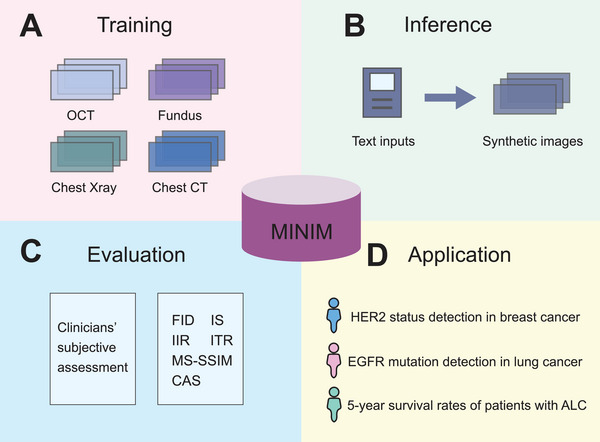
**Workflow and applications of MINIM**. (A) Medical images and their corresponding textual descriptions were integrated into a stable diffusion model for training; (B) Synthetic images were generated based on textual prompts during inference; (C) The quality of the generated images was evaluated using both subjective clinical assessments and objective quantitative metrics; (D) MINIM was applied to downstream clinical tasks, including mutation detection and survival analysis. OCT, optical coherence tomography; CT, computed tomography; FID, Fréchet inception distance; IS, inception score; IIR, image‐image retrieval; ITR, image‐text retrieval; MS‐SSIM, multi‐scale structural similarity index measure; CAS, classification accuracy score; HER2, human epidermal growth factor receptor 2; EGFR, epidermal growth factor receptor; ALC, advanced lung cancer.

One notable finding is MINIM's strong performance in image synthesis across various medical imaging modalities. Wang et al. [1] benchmarked MINIM against state‐of‐the‐art text‐to‐image generation models, including Imagen, DALLE, GigaGAN, and StyleGAN‐T. Image quality and clinical relevance were assessed using both expert evaluations and six objective metrics: Fréchet inception distance (FID), inception score (IS), multi‐scale structural similarity index measure (MS‐SSIM), classification accuracy score (CAS), image‐image retrieval (IIR), and image‐text retrieval (ITR) (Figure 1C). Expert evaluations capture qualitative aspects of image realism and clinical relevance that are difficult to quantify, while objective metrics provide quantitative, reproducible measures of image quality, diversity, and fidelity. For instance, in the ophthalmic OCT dataset, MINIM achieved an FID of 65.3, an IS of 5.7 ± 0.42, and an MS‐SSIM of 0.16 ± 0.03, suggesting improved balance among fidelity, diversity, and clinical realism. Notably, 91% of MINIM‐generated OCT images received the highest quality score from clinicians following reinforcement learning fine‐tuning.

The integration of novel AI technologies into clinical practice holds considerable promise [4]. However, a persistent question in the field of generative AI is how much synthetic data can improve the performance of predictive models. This study showed that MINIM not only generates high‐quality images but also has the potential to enhance clinical decision‐support systems through data augmentation. For diagnostic classification, models trained with a balanced mix of real and synthetic data showed marked improvements. For example, chest CT diagnostic accuracy increased from 0.58 to 0.79. In underperforming categories such as retinal vasculitis, synthetic OCT images selected by clinicians enhanced diagnostic performance. The use of synthetic data also improved medical report generation, as indicated by higher ROUGE‐L scores reflecting better alignment with expert annotations. In self‐supervised learning, pretraining on synthetic images improved downstream task performance following fine‐tuning, further demonstrating the value of synthetic data in medical AI development.

Wang et al. [1] further demonstrate the utility of synthetic images in improving classification accuracy for lung and breast cancer (Figure 1D). For example, in classifying EGFR mutation types in lung cancer using chest CT scans, a model trained solely on real data achieved a top‐1 accuracy of 81.5%. This improved to 91.2% when MINIM‐generated synthetic images were added at a 1:1 ratio, and reached 95.4% with a 5:1 synthetic‐to‐real ratio. Similarly, in HER2 status prediction from breast MRI, accuracy increased from 79.2% with real data alone to 94.0% when synthetic images outnumbered real ones by a factor of ten. These findings suggest that synthetic data generated by MINIM can improve model performance in clinically relevant tasks, ultimately supporting more precise and personalized interventions.

Recent advances in foundation models highlight the value of synthetic data for improving model performance, particularly in scenarios with limited real‐world datasets. For example, Hollmann et al. [5] introduced TabPFN, a tabular foundation model pretrained on large amounts of synthetic data. Their study demonstrates that pretraining on carefully designed synthetic datasets can enhance predictive accuracy and interpretability even when only small real datasets are available. This finding is highly relevant to MINIM's objectives, as MINIM similarly leverages synthetic data to mitigate data scarcity in medical imaging. By generating high‐quality images across multiple modalities from textual prompts, MINIM has the potential to augment limited datasets, improve downstream AI model performance, and enhance clinical interpretability.

Despite its promising results, the authors acknowledge that further validation is needed across diverse, prospective clinical settings to fully assess MINIM's generalizability and robustness. While the model significantly improves diagnostic performance, several challenges remain, such as potential overfitting, limited text‐image alignment with longer prompts, and integration into real‐world clinical workflows. Addressing these limitations through model refinement, expanded and diversified training datasets, and techniques such as adversarial training or adaptive learning will be essential.

In summary, this study represents a major advancement in the application of generative AI in healthcare. MINIM's ability to synthesize high‐quality medical images from text descriptions represents an important step toward addressing key challenges in medical imaging. By improving data availability and diversity, MINIM may support AI‐driven diagnostics, personalized treatment planning, and the development of innovative medical technologies. Looking ahead, clinical translation of such models will depend on rigorous validation, regulatory evaluation, clinician trust, effective system integration, and strong safeguards for patient safety and data privacy. Continued collaboration among researchers, clinicians, and regulators will be key to their safe and responsible implementation.

## Author Contributions

Y.C. and L.N. conceived, drafted, and revised the manuscript. All authors have read and approved the final manuscript.

## Conflicts of Interest

The authors declare no conflicts of interest.

## Ethics Statement

Not applicable.

## Data Availability

The authors have nothing to report.
